# Validity and reliability of Arabic version of the ID Pain screening questionnaire in the assessment of neuropathic pain

**DOI:** 10.1371/journal.pone.0192307

**Published:** 2018-03-15

**Authors:** Amani Abu-Shaheen, Shehu Yousef, Muhammad Riaz, Abdullah Nofal, Sarfaraz Khan, Humariya Heena

**Affiliations:** 1 Research Center, King Fahad Medical City, Riyadh, Saudi Arabia; 2 Anesthesia Department, King Fahad Medical City, Riyadh, Saudi Arabia; 3 Department of Health Sciences, University of Leicester, Leicester, United Kingdom; 4 Disaster Management Unit, King Saud University Medical City, Riyadh, Saudi Arabia; Weill Cornell Medicine-Qatar, QATAR

## Abstract

Diagnosis of neuropathic pain (NP) can be challenging. The ID Pain (ID-P) questionnaire, a screening tool for NP, has been used widely both in the original version and translated forms. The aim of this study was to develop an Arabic version of ID-P and assess its validity and reliability in detecting neuropathic pain. The original ID-P was translated in Arabic language and administered to the study population. Reliability of the Arabic version was evaluated by percentage observed agreement, and Cohen’s kappa; and validity by sensitivity, specificity, correctly classified, and receiver operating characteristic (ROC) curve. Physician diagnosis was considered as the gold standard for comparing the diagnostic accuracy. The study included 375 adult patients (153 [40.8%] with NP; 222 [59.2%] with nociceptive pain). Overall observed percentage agreement and Cohen’s kappa were >90% and >0.80, respectively. Median (range) score of ID-P scale was 3 (2–4) and 1 (0–2) in the NP group and NocP group, respectively (p<0.001). Area under the ROC curve was 0.808 (95% CI, 0.764–0.851). For the cut-off value of ≥2, sensitivity was 84.3%, specificity was 66.7%, and correct classification was 73.9%. Thus, the Arabic version of ID-P showed moderate reliability and validity as a pain assessment tool. This article presents the psychometric properties of the Arabic version of ID Pain questionnaire. This Arabic version may serve as a simple yet important screening tool, and help in appropriate management of neuropathic pain, specifically in primary care centers in the Kingdom of Saudi Arabia.

## Introduction

Clinicians are often faced with the challenge of managing patients who present with chronic pain. Accurate assessment of the type of pain is the first and the most important step for optimal management of chronic pain and improving therapeutic outcome. Chronic pain can be classified into three main categories: nociceptive pain (NocP), neuropathic pain (NP), and the coexistence of NocP and NP, *i*.*e*., mixed pain [1, 2].

The International Association for the Study of Pain (IASP) defines NP as “pain caused by a lesion or disease of the somatosensory nervous system” [3, 4]. A large number of patients are affected by chronic NP with an estimated prevalence of 6.9% to 10% [5]. Common signs and symptoms of NP include mechanical allodynia, burning sensation, electric shock sensation, hyperalgesia, etc. [6]. Patients with NP often suffer from difficulty in sleeping, lack of energy, drowsiness, and difficulty in concentrating, leading to psychological distress, physical disability, and reduced overall quality of life. As a result, NP also poses a substantial burden on the health resources [7, 8].

Unlike NP, NocP is caused by damage to body tissues leading to activation of nociceptors. Pathologically, it is distinguished from NP by a normal somatosensory nervous system and clinically by a sharp, aching, or throbbing pain [9].

A pilot study conducted by Hassan *et al*. (2004) in 10 centers in the Middle East Region reported the prevalence of NP as 41% and NocP as 59% in the patients with chronic low back pain [10]. Another multicenter study conducted in 2010 to assess the prevalence of NP among adults with lower back pain in the Arabian Gulf region found that, of the 1134 patients, 628 (55%) patients had NP. Factors associated with NP included advanced age, female gender and diabetes with or without hypertension [11].

NP does not respond to the conventional pain treatment. However, it can be managed with specific therapeutic agents such as tricyclic antidepressants, serotonin norepinephrine reuptake inhibitors, antiepileptics, opioids, and topical lidocaine. [12] Therefore, it is of utmost importance that proper evaluation of pain is carried out, so that correct diagnosis is made and appropriate treatment is facilitated. In recent years, several standardized screening tools have been developed to aid the identification and classification of NP on the basis of patient-reported verbal descriptors of pain qualities [13–15]. For diagnosis of NP, numerous NP questionnaires have also gained popularity due to their high efficiency, sensitivity, and specificity. These tools are cost-effective, do not require special equipment or examinations, and are easy to use even by those who know little about NP [13–15].

The ID Pain questionnaire (ID-P) is a six-item screening questionnaire [16]. It includes five sensory descriptor items and one item related to joint pain, which identifies NocP. The ID-P tool appears to accurately indicate the presence of neuropathic component of pain. This simple tool, which can be self-administered, could be of immense use in primary care settings [16]. The ID-P questionnaire has been translated in several languages and is widely used [16–20]. However, ID-P validation process has not been done in the Arab population. The aim of the study was to develop an Arabic version of the ID-P and to assess its validity and reliability in improving patient care.

## Materials and methods

### Study population

The study was conducted on Saudi adult patients attending pain management and diabetes clinics at King Fahad Medical City (KFMC), a tertiary hospital in Riyadh, the central region of Saudi Arabia, over a period of one year from March 2016 to March 2017. Patients suffering from moderate to severe NP or NocP pain (scoring 5 or higher on a 0–10 numerical rating scale) for at least three months were included in the study. Patients with pain of unknown origin, mixed-type pain, and diffuse pain which includes fibromyalgia syndrome, myofascial pain, complex regional pain syndrome, cancer pain, and headaches were excluded. Also, patients with substance abuse, chronic alcoholism, severe depression, as well as those incapable of understanding the questionnaire were excluded.

### Ethical consideration

The study was approved by the Institutional Review Board at KFMC, Riyadh, Saudi Arabia. Written informed consent was obtained from the participants who met inclusion criteria and agreed to participate in the study.

### Translation of the questionnaire

The adaptation procedure was monitored by a seven-member expert panel including two specialists in pain management, an expert in methodology, an expert in clinical research, and an expert in linguistics. The International Guidelines for Cross-Cultural Adaptation of Health Questionnaires and Diagnostic Tests were followed [21,22]. An internationally-accepted translation methodology was used by a well-established linguistic validation process.

The first step was the forward translation of the questionnaire to the Arabic language by bilingual translators, whose mother tongue was the Arabic. The first translator was informed about the questionnaire and its applications. The second translator was neither aware nor informed of the concepts being quantified and had no medical or clinical background. Each translator independently produced a translated version of the questionnaire, and then the two versions were synthesized into a single translation.

The second step was the backward translation into the original language, which was conducted by two individuals with the source language (English) as their mother tongue. The two translators were neither aware nor informed of the concepts explored, were from non-medical background, and were totally blind to the original version.

A meeting, which included the experts and the two translator teams (forward and back translators), was then conducted to assess the semantic and conceptual equivalence between the original and the translated versions. The expert committee’s role was to consolidate all the versions of the questionnaire and develop what was considered to be the pre-final version of the questionnaire for field-testing. All the translations were, thus, reviewed by the committee and a consensus was reached.

The final stage of adaptation process was the pretest, which involved administration of the pre-final version of questionnaire to a sample of 30 patients (other than those included in the validation sample) to detect potential conceptual problems. After completing the form, each subject was interviewed to probe his/her understanding regarding each questionnaire item and the chosen response.

### Study design

Patients were divided into two groups. First group included patients diagnosed with NP by a pain specialist in pain clinics as per the guidelines established by the IASP, [23] whereas the second study group included patients with NocP.

The Arabic version of the ID-Pain was administered twice to the study population, by the same investigator **(S1 Questionnaire).** During the first examination, the participants were interviewed to gather information about socio-demographic and clinical characteristics, and were asked to fill-in the ID-Pain questionnaire. All the subjects were re-evaluated two to four hours later for second examination. The ID-P was again filled by the subjects to assess the time stability of measurements. The assessment of the psychometric properties of the ID-Pain focused on reliability and validity.

### Sample size estimation

Sample size was calculated assuming α = 0.05 and p = 41% with power = 0.95 and maximum difference = 0.5. The sample size that is needed to validate the questionnaire for the two groups was 376 patients.

### Statistical analysis

Socio-demographic and clinical characteristics of patients were summarized for the whole sample (NP group and NocP group) using frequency (%) for categorical variables and mean (standard deviation, SD) for continuous variables. For continuous variables, normality was assessed using Shapiro Wilk test and histograms. The socio-demographic and clinical characteristics were compared between the two groups using chi-square test for categorical variables and t-test or Mann Whitney’s U test for continuous variables.

### Examining reliability of ID-P

The ID-P responses were collected in two sessions, one pre-assessment and the other post-assessment. Therefore, for test-retest reliability of the ID-P scale, the percentage observed agreement and Cohen’s kappa statistics were computed and reported. Intra-class-correlation coefficient (95% confidence interval; CI) between pre- and post-ID-P scale total scores was also computed. To check the internal consistency of the six items of the ID-P scale, Cronbach’s α was computed for the pre- and post-measures of the scale.

### Assessing validity of ID-P

Initially, median (interquartile range) of the total scores for ID-P were computed and Mann Whitney’s U test was used to compare the scores between NP and NocP groups. Thereafter, to assess the discriminant validity of ID-P in identifying the NP, we used receiver operating characteristics curve (ROC) analyses. A binary variable with physician diagnosis of (NP = 1) versus (NocP = 0) was created. With this binary variable, separate ROC analyses were conducted for the total scale scores of ID-P. These ROC analyses provided the best cut-off points of the scales total scores of ID-P with respect to the corresponding diagnostic values. For each cut-off-point of the scores, sensitivity, specificity, correctly classified, positive likelihood ratio, negative likelihood ratio, were also calculated. Based on Youden’s index [24], a best cut-off-value of the total scores of ID-P was determined, area under the ROC curve (95% CI) was computed and reported, which determined the discriminant validity (diagnostic ability) of the total scores. All these analyses were separately performed for the pre- and post-measure of the scales.

A statistical significance level of *p*<0.05 was used to reject the null hypothesis. The analyses were conducted using statistical software of SPSS software version 17.0 (Chicago, IL, USA) and Stata version 12 (StataCorp, Texas USA).

## Results

### Patient population

The study included 375 adult patients, of which 153 (40.8%; mean age: 48.4 ±12.0 years; age range: 21–71 years) patients were diagnosed with NP and 222 (59.2%; mean age: 48.9±14.5 years; age range: 18–87 years) with NocP by expert pain physicians. Demographic and clinical characteristics of the patients are depicted in **Table 1**.

**Table 1:** Comparison of the demographic and clinical characteristics of patients between neuropathic and nociceptive pain group.

Variables		Neuropathic pain (n = 153); n(%)	Nociceptive pain (n = 222); n(%)	p-value
Gender	Male	51 (33.3)	58 (26.1)	0.131
Education Level	High school and above	77 (50.3)	133 (59.9)	0.066
Occupation	Employed versus Unemployed	49 (32.0)	46 (20.7)	**0.016**
Marital Status	Married	136 (88.9)	176 (79.3)	**0.012**
	Single	13 (8.5)	24 (10.8)	
	Widow/Divorced	4 (2.6)	22 (9.9)	
Medication use	**-**	126 (82.4)	119 (53.6)	**<0.001**
Comorbidities	Diabetes mellitus	60 (39.2)	65 (29.3)	**0.045**
	Hypertension	54 (35.3)	62 (27.9)	0.129
	Liver disease	6 (3.9)	3 (1.4)	0.168
	Heart disease	21 (13.7)	15 (6.8)	**0.024**
	Lung disease	8 (5.2)	6 (2.7)	0.205
	Kidney disease	8 (5.2)	10 (4.5)	0.747
	Physical exercise	44 (28.8)	41 (18.6)	**0.021**
Age	Years; Mean (SD)	48.4 (12.0)	48.9 (14.5)	0.721
Height	cm; Mean (SD)	160.5 (10.3)	158.3 (9.2)	**0.045**
Weight	Kg; Mean (SD)	82.0 (17.1)	78.9 (18.5)	0.138
Body mass index	Kg/m^2^; Mean (SD)	32.0 (7.1)	31.6 (7.7)	0.665
Duration of pain	Month*; Mean (SD)	48 (24–84)	24 (12–48)	**0.002**

Significant odds ratios (OR) are in bold

The majority of NP patients had low back pain/referred pain (n = 74; 48.4%), diabetic polyneuropathy (n = 16; 10.5%), and lumbo-sciatic pain (n = 12; 7.8%), whereas, the majority of NocP patients had osteoarthritis (n = 111; 50.0%), inflammatory chronic arthritis (n = 41; 18.5%) and mechanical low back pain (n = 21; 9.5%) (**Table 2**).

**Table 2:** Etiology of pain in the two study groups.

Etiology	Neuropathic pain	Nociceptive pain
Low back pain/Referred Pain	74 (48.4)	0 (0.0)
Diabetic polyneuropathy	16 (10.5)	0 (0.0)
Lumbosciatic pain	12 (7.8)	0 (0.0)
Peripheral neuropathy	8 (5.2)	0 (0.0)
Injury	7 (4.6)	0 (0.0)
Diabetes Painful Neuropathic Pain	3 (2.0)	0 (0.0)
Post Herpetic Neuralgia	2 (1.3)	0 (0.0)
Chronic polyradiculopathies	2 (1.3)	0 (0.0)
Trigeminal Neuralgia	1 (0.7)	0 (0.0)
Osteoarthritis	0 (0.0)	111 (50.0)
Inflammatory chronic arthritis	0 (0.0)	41 (18.5)
Mechanical low back pain	0 (0.0)	21 (9.5)
Myofascial pain	0 (0.0)	5 (2.3)
Central Neuropathic pain	0 (0.0)	1 (0.5)
Others	28 (18.3)	43 (19.4)

The top three painkillers that were used by NP patients were paracetamol (n = 71; 46.4%), meloxicam (n = 35; 22.9%), and diclofenac sodium (n = 35; 22.9%). In the NocP group, 56 (25.2%) patients used paracetamol, whereas diclofenac sodium and meloxicam were used by 40 (18.0%) and 34 (15.3%) patients, respectively.

### Reliability of the scale

The observed percentage agreement and Cohen’s kappa statistics computed for examining the test-retest reliability of the pre-post assessment of the items of the ID-P scale ranged from ‘limited to joints: 93.6%, CK = 0.872’ to ‘feel like hot/burning: 97.9%, CK = 0.956’ in the overall sample; while, it ranged from ‘feel like pins and needles: 95.4%, CK = 0.886’ to ‘feel like hot/burning: 97.4, CK = 0.944’ in the NP group (**Table 3**). The intra-class correlation coefficient (ICC) between pre- and post-ID-P scale total scores for the overall sample was 0.979 (95% CI, 0.974–0.982), whereas, for NP and NocP groups, the ICC were 0.960 (95% CI, 0.944–0.971), and 0.976 (95% CI, 0.969–0.982), respectively. Cronbach’s α for the pre-assessment were 0.506, 0.354, and 0.446 for the overall sample, NP, and NocP respectively. Similar values of the Cronbach’s α were observed for the post-assessment measures of the ID-P scale.

**Table 3:** Test-retest reliability (agreement between pre- and post-assessment of ID-P response) and its internal consistency.

Items of the ID-P scale	Overall sample		Neuropathic pain		Nociceptive pain	
	Agreement n (%)	Cohen’s Kappa	Agreement n (%)	Cohen’s Kappa	Agreement n (%)	Cohen’s Kappa
Feel like pins and needles	259 (95.7)	0.901	146 (95.4)	0.886	213 (96.0)	0.910
Feel like hot/burning	367 (97.9)	0.956	149 (97.4)	0.944	218 (98.2)	0.956
Feel numb	360 (96.0)	0.920	148 (96.7)	0.915	212 (95.5)	0.906
Feel like electric shocks	360 (96.0)	0.919	145 (94.8)	0.890	215 (96.9)	0.931
Made worse with the touch of clothing or bed sheets	366 (97.6)	0.932	149 (97.4)	0.937	217 (97.8)	0.924
Limited to your joints	351 (93.6)	0.872	147 (96.1)	0.738	210 (94.6)	0.864
Total scale score: test-retest reliability	**ICC (95% CI)**		**ICC (95% CI)**		**ICC (95% CI)**	
	0.979 (0.974–0.982)		0.960 (0.944–0.971)		0.976 (0.969–0.982)	
Consistency of the above 6 items scale	**Cronbach’s α**		**Cronbach’s α**		**Cronbach’s α**	
Pre-assessment	0.506		0.354		0.446	
Post-assessment	0.531		0.272		0.461	

**Agreement:** Observed agreement of the items of ID-P between pre- and post-assessment.

**Cohen’s Kappa:** Statistics for assessing test-retest reliability of each items of the scale (categorical).

**ICC (95% CI):** Intra class correlation coefficient (95% confidence interval) for examining test-retest reliability of the scale total score (continuous variable).

**Cronbach’s α (alpha):** Coefficient used to examine internal consistency of the ID-P scale

### Validity of the scale

**Fig 1:**
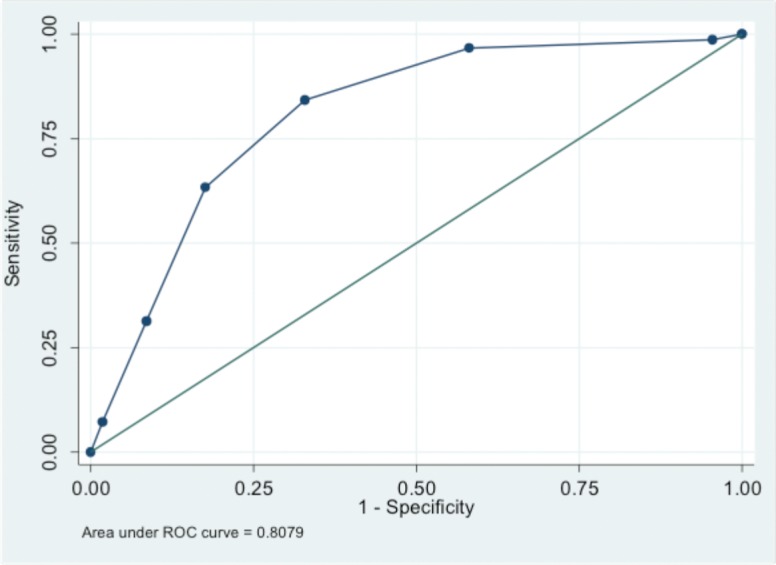
ROC curve analysis: Plot of sensitivity versus 1-specificity (ID-P scale).

The medians (interquartile range) for the total score of ID-P scale were 3 (range, 2–4) and 1 (range, 0–2) in the NP group and NocP group, respectively (*p*<0.001). Using the physician (clinical) diagnosis (NP versus NocP) as the gold standard, when the discriminant validity of the ID-P scale was examined using the receiving operating characteristics (ROC) curve analyses, the area under the ROC curve was estimated to be 0.808 (95% CI, 0.764–0.851) for ID-P total score (**Fig 1**).

Further discriminant statistics (*i*.*e*., percent sensitivity, specificity, correct classification, positive and negative likelihood ratio) for each of the possible cut-off values of the ID-P total scores are presented in **Table 4**. For the cut-off value of ≥2, the sensitivity was 84.3%, the specificity was 66.7% and the correct classification was found to be 73.9%. The positive likelihood ratio and negative likelihood ratio were found to be 2.529 and 0.235, respectively. The adjusted odds ratio of the clinical diagnosis of the NP for the per unit increase in the total score of ID-P was 2.58 (95% CI, 2.02–3.28), *p*<0.001 (**Table 5**).

**Table 4:** Discriminative characteristics of the total score for ID-P scale in identifying patients with neuropathic pain versus nociceptive pain (ROC curve analysis).

Cut-point	Sensitivity (%)	Specificity (%)	Correctly Classified (%)	LR+	LR-
(≥-1)	100.0	0.0	40.8	1.000	
(≥ 0)	99.4	4.1	42.9	1.035	0.161
(≥ 1)	96.7	39.6	62.9	1.603	0.082
**(≥ 2)**	**84.3**	**66.7**	**73.9**	**2.529**	**0.235**
(≥ 3)	62.1	82.4	74.1	3.534	0.460
(≥ 4)	30.7	91.4	66.7	3.589	0.758
(≥ 5)	8.5	99.1	62.1	9.431	0.923
(≥ 5)	0.0	100.0	59.2		1.000

Area under receiving operating characteristics (ROC) curve = 0.808 (95% CI, 0.764–0.851)

**Cut-point:** cut-off-value of the ID-P total scale score.

**LR+:** Positive likelihood ratio

**LR-:** Negative likelihood ratio

**Table 5:** Logistic regression models to adjust the effect of total scale score in predicting the physician diagnosis of neuropathic pain.

Variables	ID-P scale; OR (95% CI)	p-values
**Total scores**	2.58 (2.02, 3.28)	<0.001
Employed vs. Unemployed	2.67 (1.27, 5.59)	0.009
Marital Status	0.56 (0.31, 0.99)	0.048
Medication use	0.30 (0.15, 0.60)	0.001
Diabetes mellitus	0.75 (0.38, 1.47)	0.396
Heart disease	0.66 (0.25, 1.77)	0.410
Physical exercise	0.36 (0.18, 0.73)	0.005
Height (cm)	1.02 (0.99, 1.06)	0.145
Duration of Pain (month)	1.01 (1.00, 1.01)	0.074

## Discussion

The main advantages of the six-item ID-P questionnaire include its extreme brevity (takes only few minutes to complete) and simplicity (does not require any physical examination or assistance from physicians). Moreover, it has been found to be of high sensitivity and specificity [16]. Thus, ID-P can serve as an important tool for general practitioners in the initial screening of patients with chronic pain. Depending on the screening results, further assessment can be carried out, which may subsequently influence management decisions.

In the present study, Arabic version of ID-P was developed to assess its validity and reliability in improving patient care. In order to ascertain that the translated version accurately reflected the original one, a robust approach was followed including a panel of experts and bilingual translators. All the versions were then considered by the expert panel to formulate the final version. Additionally, a pretest was carried out to ensure that the adapted version retained its equivalence in an applied situation. The results of the present study demonstrated that the Arabic version of ID-P is a reliable and efficient method to detect NP. Percentage agreement and Cohen’s kappa statistics showed that there was a high test-retest reliability of the scale in the overall sample as well as in the two sub-groups. The intra-class correlation coefficient between pre- and post-ID-P scale total scores also showed a high level of test-retest reliability. Further, an area under the ROC curve of 0.808 (95% CI, 0.764–0.851) indicated a good diagnostic value. Most importantly, the results showed a good sensitivity and moderate specificity of the Arabic version of ID-P in evaluating patients with chronic pain.

Previous translated and adapted versions of ID-P have also been validated as useful screening tools [17–20]. A cross-sectional validation study conducted on 145 (51.2%) subjects with NP and 138 (48.8%) with NocP reported the Spanish version of ID-P as a practical, convenient, and reliable tool for self-assessment of NP. It was found to be time stable (test-retest r-Pearson = 0.98; *p*<0.0005) with a cut -off value of ≥3 points, area under the ROC curve of 0.89; *p*<0.0005, sensitivity value of 0.81, specificity of 0.84, and kappa coefficient of agreement with reference clinical diagnosis of 0.65 [17]. Another study conducted on 100 patients (NP, n = 24; NocP, n = 49; mixed pain, n = 27) reported the Thai version of ID-P as an efficient method to diagnose NP with a good predictive value (83% sensitivity, 80% specificity, area under the ROC curve 0.890; 95% CI, 0.824–0.955) [19]. In our study, corresponding to a total score of ID-P (cut-off) ≥2, the Youden’s index [24] of (0.51) is maximum, but majority of the studies [16–20] has recommended a cut-off value of ≥3 which is consistent with a Youden’s index of (0.45) in this study. These two indices are very close, and hence a cut-off value ≥2 or 3 is recommended for this population. The slight variation of our results could be explained by the difference of population in terms of ethnicity, cultural and educational background.

The high sensitivity and moderate specificity achieved in the Arabic version of the ID-P may help in delineating the specific NP component in patients and differentiate them from those suffering from NocP. In the Kingdom of Saudi Arabia, given the setting of primary health care centers in far-flung areas, it is possible that patients with chronic pain are prescribed inappropriate medication owing to lack of availability of pain specialists. In such a scenario, ID-P can be used by a general practitioner to correctly classify the type of pain and institute appropriate treatment. The strength of the present study lies in the fact that the Arabic version of the questionnaire can be used by the patient himself, without any prior physical examination and without consulting any pain physician. The present study observed all the principles of validation. Pain physicians were involved in the study to objectively assess the pain type through history and examination. They were also involved to compare the translated versions of the screening tools. The testing of the subjective assessment of a patient’s pain cannot, of course, supersede the physician assessment. However, it can lead to better measures for screening and subsequent pain management.

We used the gold standard of diagnosis based on clinical judgment by different pain specialists. However, recent recommendations among clinicians to diagnose pain with well-defined criteria were not applied and we could not account for the variation of precise diagnosis in the type of pain.

In addition, a large scale epidemiologic study in the community is warranted to further substantiate the results of this study. Involvement of different centers could not be possible because of the sheer logistics and resources involved in multicenter projects. Ideally, future research should also address issue of reaching clinical consensus on signs and symptoms of NP and its improved understanding for better diagnosis and treatment. This would pave a way for the development of mechanism-based therapies.

In summary, the Arabic version of ID-P assessed in the present study had an excellent test-retest reliability, and internal consistency of ID-P was unacceptable consistency. The face and content validity, values for sensitivity, specificity and correct classification were also comparable with the previously suggested cut-off values [17–20]. Hence, this study showed that the Arabic version ID-P is reliable when used for the diagnosis of NP in Arabic patients.

Numerous pain diagnostic questionnaires are available for the assessment of NP. ID-P has been used widely as an original version as well as in translated forms. The Arabic version of ID-P has moderate reliability and validity as a pain assessment tool. The present study provides the rationale to encourage extension of its use by general physicians to promote appropriate pain screening in patients suffering with chronic pain. This also suggests that there is a need for the development of local diagnostic questionnaires suited to the Arab population.

## Supporting information

S1 Questionnaire ID pain questionnaire. (PUB)
